# 

**Published:** 1888-12

**Authors:** 


### HALL'S JOURNAL OF HEALTH

TRUTH DEMANDS NO SACRIFICE; ERROR CAN MAKE NONE.

VOL. 35. DECEMBER, 1888. No 12.

### LAST OF THE SERIES OF 1888.

The present issue completes the current year series of this Journal. It was established thirty-five years ago by the late distinguished Dr. W. W. Hall, in the interest of good health by right living, nor has it since failed in a single instance, to make its customary monthly visit to its patrons.

As in the past, the publication will be continued in the interest of its patrons as to all matters relating to their physical well being, holding as we do, that whatever is important that one should know in this behalf, it is fit that all should know. There should be no monopoly if the methods of self preservation, nor the cure of disease, nor shall we fail to present any fact which bears upon the subject merely because of its unpopularity. A journal governed by such a tame policy would not only be of no value to its readers, but a downright hinderence to them, inasmuch as a perfect agreement between individuals, offers no inducement to mental growth.

The self-satisfied individual is always a non-progressive one. To such a new thought or a strange idea, is a thing to be shunned as a disturber of the peace, and if sufficiently alive to quarrel with anything, it is with his newspaper or magazine, for imposing upon him the necessity of thinking in an unfamiliar direction. We do not court such readers, nor do we expect to maintain agreeable relations with them, since our endeavors are less for profit than to accomplish a good work, in which purpose we do not mean to fail.

As our next issue will commence the 35th volume, we desire to call attention once more to the two advertised premium books, either one is worth the entire yearly subscription price of our Journal, viz: the FARM and HOUSEHOLD CYCLOPÆDIA and the CYCLOPÆDIA of USEFUL KNOWLEDGE. The choice of these is offered with the Journal for twelve months, for $1.25, or for six months for $1, when remitted directly to this office.

Will not each of our patrons make a little effort among friends, to procure us one additional subscriber ? We ought to number at least, 25,000. During the past week there has been a little delay in mailing these books to new subscribers, owing to the great demand for them having exhausted the edition, but a new edition has been provided in numbers which will preclude the likelihood of such a recurrence. May the coming year be one of great prosperity and happiness to our readers one and all.

### BOOK NOTICES.

### THE BEST OF THEIR KIND.

BABYLAND.—Suited to lisping juveniles.

OUR LITTLE MEN AND WOMEN, for youths of a tender age. D. Lothrop Co., Boston.

YOUTH'S COMPANION.—Instructive as well as entertaining for all ages. Perry Mason & Co., Boston.

THE CENTURY contains original articles from the pen of many of our more distinguished writers, among which are the Life of Abraham Lincoln and Siberia now being continued, alone worth the year's subscription. The Century Co., New York City.

VICK'S ILLUSTRATED MONTHLY, devoted to flowers and garden products. James Vick, Rochester, N. Y.

HORTICULTURAL ART JOURNAL.—Elegantly illustrated and ably conducted. Stecher Lithographic Co., Rochester, N. Y.

TABLE TALK.—What to eat and how to cook it. Philadelphia, Pa.

Our thanks are due for the following named essays and treatises:

EDUCATION IN BAVARIA, by Sir Philip Magnus.

THE PREFERABLE CLIMATE FOR PHTHISIS by Prof. Charles Dinison, A. M. M. D..

Sixteenth annual meeting American Public Health Association, 1888, Milwaukee.

CATARACT EXTRACTIONS and RELIEF OF HEADACHE and EYE PAINS, by Prcf. Julian J. Chisolm, M. D.

TRANSACTIONS: AMERICAN ASSOCIATION of OBSTETRICIANS and GYNECOLOGISTS, held in Washington, D. C. September 1888.

MINERAL and THERMAL SPRINGS of CALIFORNIA, by Prof. W. F. McNutt, M. D., San Francisco.

TREATMENT of PERITONITIS, etc., by Prof. L. S. McMurtry, M. D.

TREATMENT of STRICTURE, by G. C. H. Meier, M. D., New York City.

THE SLÖJD in the SERVICE of the SCHOOL, translated from the German by Wm. H. Carpenter, Ph. D., New York Educational Association.

DRIFT of the AGE. Pith of the Dix Lenton Lecture, illustrated.

TWENTY-FOURTH ANNUAL REPORT of the "SHELTERING ARMS," New York.

EXPERIMENTAL RESEARCHES IN RELATION OF DRESS TO PELVIC DISEASES OF WOMEN, by Prof. J. H. Kellogg, M. D.

WHEN AGE GROWS YOUNG, a romance by Hyland C. Kirk. Charles T. Dillingham 720 Broadway publisher. This is a very singular production, with a single incident of clairvoyance, a weak discussion of the spiritual theory and a ghost prowler that proves to be a cave dweller in a deserted mine. We can make nothing of it but a hotch-potch of absurdity, and bad morals on the part of some of its leading characters. We can no more review it than we can rearrange the scattered particles of a grotesque mosaic. As a puzzle for the curious, it may be amusing, if not instructive.

EATING FOR STRENGTH, or Food and Diet in their relatinn to HEALTH and WORK. By M. L. Holbrook, M. D.—M. L. Holbrook & Co. New York, 1888. This handy little volume covers the whole dietetical field with valuable table analyses of the nutrient value of foods best adapted to sustaining life. As a simple guide to health suited to all ages and conditions. this work will be found of great importance. There are also a large number of cooking recipes of more value to the household, than the cost of the work.

